# iDNS3IP: Identification and Characterization of HCV NS3 Protease Inhibitory Peptides

**DOI:** 10.3390/ijms26115356

**Published:** 2025-06-03

**Authors:** Hui-Ju Kao, Tzu-Hsiang Weng, Chia-Hung Chen, Chen-Lin Yu, Yu-Chi Chen, Chen-Chen Huang, Kai-Yao Huang, Shun-Long Weng

**Affiliations:** 1 Department of Medical Research, Hsinchu MacKay Memorial Hospital, Hsinchu City 30071, Taiwan; 2 Department of Medical Research, Hsinchu Municipal MacKay Children’s Hospital, Hsinchu City 30068, Taiwan; 3 Department of Obstetrics and Gynecology, MacKay Memorial Hospital, Taipei City 10449, Taiwan; 4 Department of Medicine, MacKay Medical College, New Taipei City 25245, Taiwan; 5 Institute of Biomedical Sciences, MacKay Medical College, New Taipei City 25245, Taiwan; 6 Department of Biological Science and Technology, National Yang Ming Chiao Tung University, Hsinchu City 30068, Taiwan; 7 Department of Obstetrics and Gynecology, Hsinchu MacKay Memorial Hospital, Hsinchu City 30071, Taiwan; 8 Department of Obstetrics and Gynecology, Hsinchu Municipal MacKay Children’s Hospital, Hsinchu City 30068, Taiwan

**Keywords:** hepatitis C virus (HCV), NS3 protease inhibitory peptides, antiviral peptides, therapeutic peptides, machine learning, amino acid composition, peptide prediction, web server

## Abstract

Hepatitis C virus (HCV) infection remains a significant global health burden, driven by the emergence of drug-resistant strains and the limited efficacy of current antiviral therapies. A promising strategy for therapeutic intervention involves targeting the NS3 protease, a viral enzyme essential for replication. In this study, we present the first computational model specifically designed to identify NS3 protease inhibitory peptides (NS3IPs). Using amino acid composition (AAC) and K-spaced amino acid pair composition (CKSAAP) features, we developed machine learning classifiers based on support vector machine (SVM) and random forest (RF), achieving accuracies of 98.85% and 97.83%, respectively, validated through 5-fold cross-validation and independent testing. To support the accessibility of the strategy, we implemented a web-based tool, iDNS3IP, which enables real-time prediction of NS3IPs. In addition, we performed feature space analyses using PCA, t-SNE, and LDA based on AAindex descriptors. The resulting visualizations showed a distinguishable clustering between NS3IPs and non-inhibitory peptides, suggesting that inhibitory activity may correlate with characteristic physicochemical patterns. This study provides a reliable and interpretable platform to assist in the discovery of therapeutic peptides and supports continued research into peptide-based antiviral strategies for drug-resistant HCV. To enhance its flexibility, the iDNS3IP web tool also incorporates a BLAST-based similarity search function, enabling users to evaluate inhibitory candidates from both predictive and homology-based perspectives.

## 1. Introduction

Hepatitis C virus (HCV) continues to pose a significant global health challenge, as emphasized by the World Health Organization (WHO) [1,2]. Affecting millions worldwide, HCV infection can lead to severe complications, including cirrhosis and hepatocellular carcinoma. According to a recent global analysis, the estimated global prevalence of HCV infection in 2020 was 0.7% (95% CI 0.7–0.9), corresponding to millions of individuals at risk of liver-related outcomes. In the same year, viral hepatitis B and C together caused approximately 1.1 million deaths, reflecting a mortality burden comparable to that of tuberculosis [3,4,5,6].

Despite significant advancements in treatments, particularly with direct-acting antivirals (DAAs) targeting viral enzymes such as the NS3/4A protease, their long-term effectiveness is undermined by the emergence of drug-resistant variants and adverse effects [7,8,9,10,11,12]. These limitations highlight the urgent need for novel therapeutic strategies against HCV. The NS3 protease plays a central role in the HCV life cycle by cleaving the viral polyprotein precursor into essential components, including NS3, NS4A, NS4B, NS5A, and NS5B, all required for replication and assembly [13,14,15,16]. Its indispensable role makes the NS3 protease an attractive target for therapeutic development [13,17]. Clinically approved NS3 inhibitors such as Glecaprevir and Voxilaprevir have shown strong efficacy [18,19]; however, their use has led to the emergence of resistance-associated variants that limit their long-term utility [20].

Peptide-based NS3 protease inhibitors have drawn increasing attention due to their high specificity, low toxicity, and capacity to target conserved regions within the protease. These features potentially allow them to bypass common resistance mechanisms. Recent studies have demonstrated that antiviral peptides can inhibit viral proteases, including the NS2B/NS3 protease of Zika virus [21], indicating their versatility and therapeutic potential. This suggests that similar peptide-based strategies could be effective in inhibiting the HCV NS3 protease.

Developing a predictive model for NS3 protease inhibitory peptides (NS3IPs) offers a promising avenue to accelerate the discovery of new therapeutic agents. Antiviral peptides are known to inhibit viral enzymes with a high specificity and minimal off-target effects, and they have been successfully applied to proteases from HIV, HCV, and Zika virus [21,22]. These findings underscore the importance of computational tools that facilitate the discovery of inhibitory peptides, particularly for challenging targets like the HCV NS3 protease. Several machine learning-based platforms, such as AVPpred [23], AVP-IC50Pred [24], and Meta-IAVP [25], have demonstrated the utility of computational approaches in the prediction of antiviral peptides. However, these tools are not specifically tailored to NS3 protease inhibition. For instance, AVPpred uses support vector machines (SVMs) [26] to predict general antiviral peptides; AVP-IC50Pred applies regression-based methods to estimate peptides’ potency; and Meta-IAVP uses ensemble learning for improved classification. While these platforms represent significant progress, their lack of a NS3-specific focus highlights the need for a specialized computational model.

In this study, we introduce the first dedicated machine learning model for predicting NS3 protease inhibitory peptides. By integrating sequence-based features, such as amino acid composition (AAC) and K-spaced amino acid pair composition (CKSAAP), and applying SVM and random forest (RF) [27] algorithms, the model demonstrates a high accuracy validated by 5-fold cross-validation and independent testing. To promote broader accessibility and practical use, we developed an online prediction tool, iDNS3IP (http://mer.hc.mmh.org.tw/iDNS3IP/, accessed on 1 May 2025), which provides a convenient platform for real-time NS3IP prediction. This study addresses a key gap in antiviral peptide research and contributes to ongoing efforts in advancing peptide-based therapeutics against HCV.

## 2. Results

### 2.1. Dataset Overview

The dataset used in this study consisted of 199 NS3 protease inhibitory peptides (NS3IPs) and 1010 non-inhibitory peptides (non-NS3IPs), collected and curated from publicly available experimental sources. The positive set included peptides experimentally validated to inhibit HCV NS3 protease activity, while the negative set comprised peptides with no reported inhibitory effects. As shown in Table 1, the dataset is moderately imbalanced but remains suitable for supervised classification tasks, particularly when evaluated using metrics such as balanced accuracy and AUC.

### 2.2. Amino Acid Composition Analysis of NS3 Protease Inhibitory Peptides

**Figure 1:**
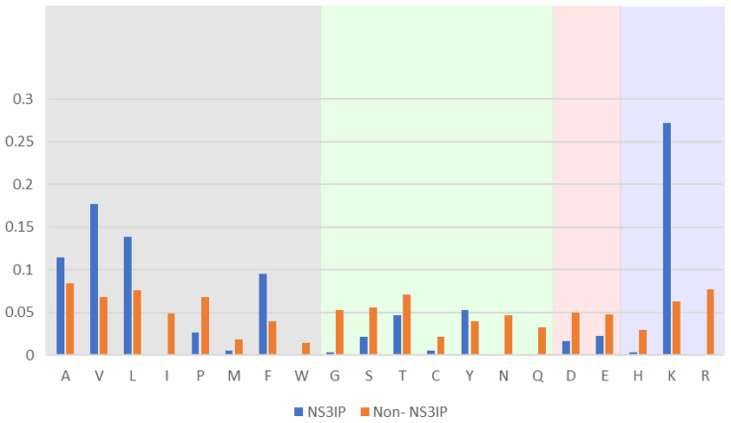
Comparison of amino acid composition between NS3 protease inhibitory peptides (NS3IPs) and non-NS3IPs. Relative frequencies of each amino acid are shown for NS3IPs (blue bars) and non-NS3IPs (orange bars). Amino acids are grouped by physicochemical properties: non-polar hydrophobic (gray background), polar uncharged (green), acidic (red), and basic (blue).

To investigate the compositional differences between NS3 protease inhibitory peptides (NS3IPs) and non-inhibitory peptides, we performed an amino acid composition analysis. As shown in Figure 1, NS3IPs exhibit a distinctive enrichment in specific residues, including hydrophobic amino acids such as valine (V) and leucine (L) and positively charged residues such as lysine (K). These residues are known to contribute to peptide–protein interactions via hydrophobic contacts and electrostatic forces, potentially supporting the inhibitory activity of NS3IPs [28].

**Figure 2:**
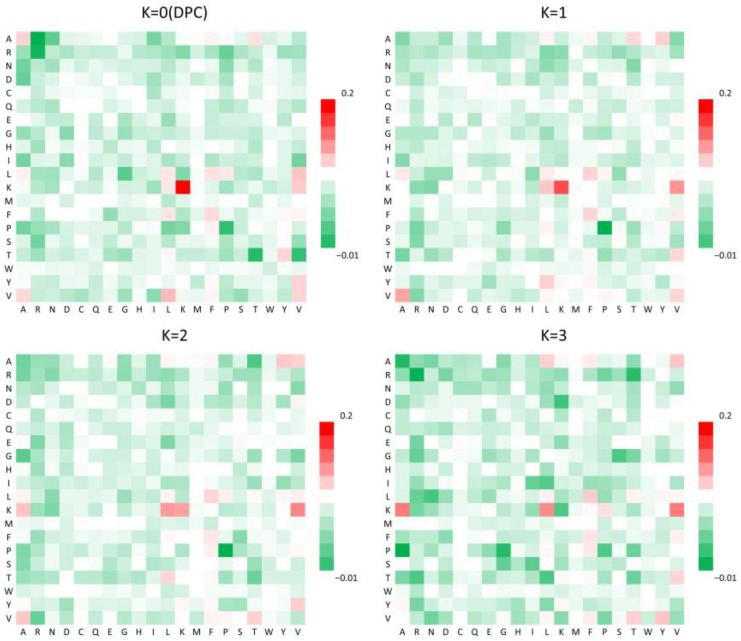
Heatmap comparison of the occurrence frequencies of 20 × 20 amino acid pairs separated by kK residues in NS3 protease inhibitory peptides (NS3IPs) and non-NS3IPs. Each matrix corresponds to a specific k value, with red indicating higher pair frequencies and green indicating lower frequencies.

In addition, a K-spaced amino acid pair composition (CKSAAP) analysis was performed to further assess sequence-level patterns. The heatmap in Figure 2 highlights the enrichment of specific amino acid pairs in NS3IPs, particularly those involving lysine (K) and valine (V). These combinations may enhance the binding affinity to the NS3 protease active site and support inhibitory function, aligning with their known roles in protein–ligand interactions [29].

**Figure 3:**
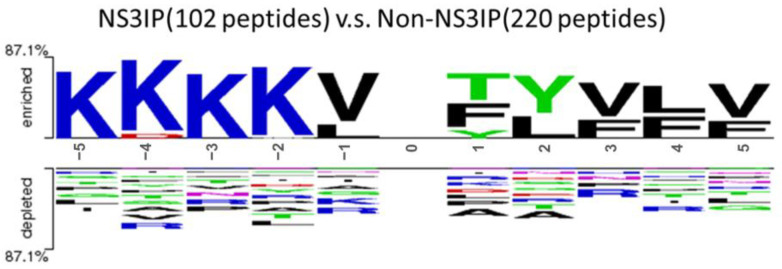
Position-specific amino acid composition in the N-terminal (positions –5 to –1) and C-terminal (positions +1 to +5) regions of NS3 protease inhibitory peptides (NS3IPs) compared with non-NS3IPs.

To complement this analysis, we used Two Sample Logo plots to examine residue preferences in the flanking regions of the peptides. Figure 3 shows the N-terminal (−5 to −1) and C-terminal (+1 to +5) positional residue distributions, revealing the consistent enrichment of lysine (K) at positions −5 to −2 among NS3IPs. This positional preference may reflect a functional role in enhancing NS3 protease binding and inhibition [30].

These sequence-based features collectively demonstrate characteristic compositional patterns that distinguish NS3IPs from non-inhibitory peptides, providing important indicators for identifying potential inhibitors.

### 2.3. Physicochemical Properties of NS3IPs vs. Non-NS3IPs

To further characterize the molecular distinctions between NS3 protease inhibitory peptides (NS3IPs) and non-inhibitory peptides (non-NS3IPs), we analyzed several physicochemical properties, including polarity, net charge, hydrophobicity, and functional group composition. These descriptors were derived from the AAindex database [31] and offer insights into the chemical tendencies that may influence inhibitory activity.

**Figure 4:**
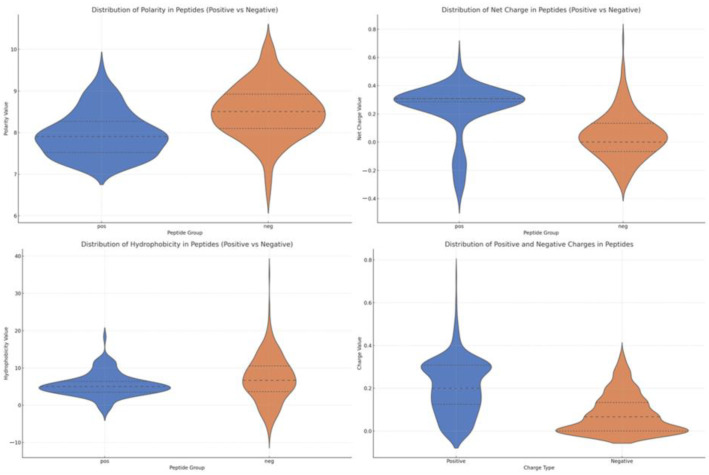
Comparison of physicochemical property profiles between NS3 protease inhibitory peptides (NS3IPs) and non-NS3IPs. Violin plots show the distributions of polarity, net charge, hydrophobicity, and the balance of positive and negative charges. For polarity, net charge, and hydrophobicity, NS3IPs are shown in blue and non-NS3IPs in orange. In the bottom-right plot, blue indicates positive charges, and orange indicates negative charges.

Polarity and net charge are critical parameters for electrostatic interactions, particularly with the negatively charged NS3 protease active site. As shown in Figure 4 (top right), NS3IPs display a more concentrated polarity distribution and a significantly higher net charge than non-NS3IPs. Additionally, Figure 4 (bottom right) reveals that NS3IPs contain a lower proportion of negatively charged residues, further supporting their compatibility with the protease’s electrostatic environment.

Hydrophobicity, another key determinant in peptide–protein binding, was examined to assess solubility and potential for interaction with the target. Figure 4 (bottom left) shows that NS3IPs are generally less hydrophobic compared to non-NS3IPs, indicating a favorable balance that preserves solubility while maintaining the ability to interact with hydrophobic pockets of the NS3 protease.

**Figure 5:**
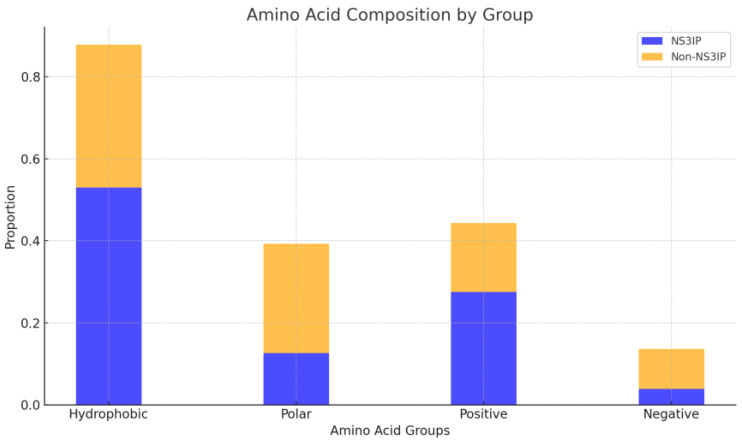
Amino acid composition by functional group in NS3 protease inhibitory peptides (NS3IPs) and non-NS3IPs. The stacked bar plot compares the proportions of hydrophobic, polar, positively charged, and negatively charged residues between NS3IPs (blue) and non-NS3IPs (orange).

To complement the sequence-based profiles, we analyzed the distribution of amino acid classes—hydrophobic, polar, positively charged, and negatively charged residues. As shown in the stacked bar chart ofin Figure 5, NS3IPs demonstrate an enrichment in hydrophobic residues such as valine (V) and leucine (L), along with positively charged residues, including lysine (K) and arginine (R). In contrast, non-NS3IPs show a higher presence of polar and negatively charged residues, which may hinder effective interactions with the protease.

These observations highlight distinct physicochemical characteristics in NS3IPs that may play a role in protease binding, suggesting that properties such as net charge and residues’ class composition could serve as distinguishing features for predictive modeling.

### 2.4. Evaluation and Feature Importance Analysis of NS3IP Predictive Models

**Figure 6:**
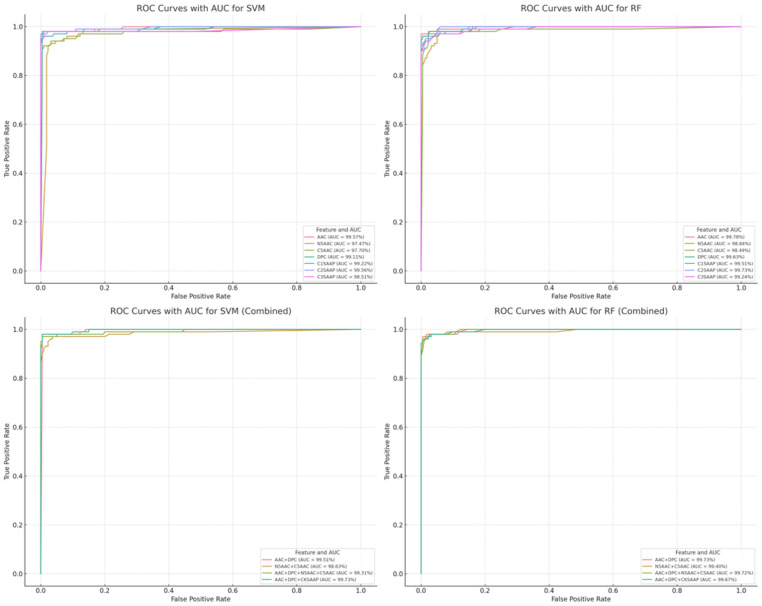
Receiver operating characteristic (ROC) curves of SVM and RF models trained with individual and hybrid feature sets. The top row shows the performance of models using individual feature sets, with SVM on the left and RF on the right. The bottom row displays the corresponding results for models trained on hybrid features. Area under the curve (AUC) values are indicated for each feature.

The predictive performance of the NS3 protease inhibitory peptide (NS3IP) classification models was assessed using five repetitions of 5-fold cross-validation for both support vector machine (SVM) and random forest (RF) classifiers. As summarized in Table 2, models trained on amino acid composition (AAC) features demonstrated a high predictive accuracy. The AAC-based SVM model achieved an accuracy of 98.85%, a balanced accuracy (B.ACC) of 97.35%, and an area under the receiver operating characteristic curve (AUC) exceeding 0.99 (Figure 6, top row). The RF model also yielded a strong performance, with an accuracy of 97.83% and a B.ACC of 96.78%. Detailed metrics for all individual feature sets and classifiers are available in Supplementary Table S1.

To examine the additive effect of combining multiple feature types, hybrid models were constructed using AAC, K-spaced amino acid pair composition (CKSAAP), and physicochemical properties. As shown in Table 3, hybrid feature models exhibited further improvements. The SVM model trained on the combined feature set achieved a 98.70% accuracy, a 98.31% B.ACC, and an AUC of 0.9973 (Figure 6, bottom row). These results are detailed in Supplementary Table S2.

**Figure 7:**
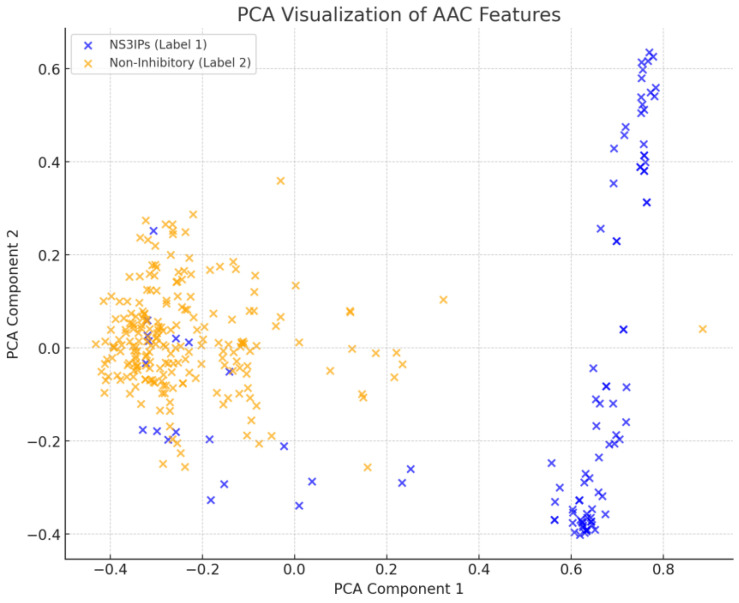
Principal component analysis (PCA) of amino acid composition (AAC) features. The scatter plot shows the distribution of NS3 protease inhibitory peptides (NS3IPs, blue) and non-inhibitory peptides (orange) along the first two principal components.

A principal component analysis (PCA) of AAC features was performed to visualize the separation between NS3IPs and non-inhibitory peptides. As shown in Figure 7, partial clustering was observed, with NS3IPs showing directional separation along the first principal component. However, some overlap in the central region indicates the presence of non-linear patterns not fully captured by linear methods.

Feature selection was also guided by considerations of computational efficiency. AAC, with its 20-dimensional feature space, offers a significantly lower computational cost than higher-dimensional descriptors such as CKSAAP or Dipeptide Composition (DPC), which contain over 400 features. This compact representation allows faster training and real-time prediction capabilities, while maintaining a high classification performance. As shown in Supplementary Table S1, the AAC feature set consistently performed well across all classifiers, demonstrating a favorable balance between predictive power and computational efficiency.

### 2.5. Robustness Validation and Online Tool for NS3IP Prediction

To assess the robustness and generalizability of the proposed models, independent testing was conducted on an external dataset. As shown in Table 4, both SVM and RF classifiers achieved an outstanding predictive performance, with accuracy, sensitivity, specificity, balanced accuracy (B.ACC), and Matthews correlation coefficient (MCC) all reaching 100%. These results confirm the reliability and stability of the AAC-based models, even when applied to unseen data.

To promote accessibility and user adoption, we developed a web-based prediction platform named iDNS3IP, which integrates both SVM and RF models for NS3IP identification. The tool is available online at http://mer.hc.mmh.org.tw/iDNS3IP/, accessed on 1 May 2025, providing users with a straightforward interface to submit peptide sequences and receive real-time predictions of their inhibitory potential. The platform supports dual-model selection, allowing users to compare outcomes from both classifiers, thereby offering flexibility in the model’s application based on user preference.

In addition to the model’s robustness, the computational efficiency of the AAC-based framework was benchmarked. With only 20 features, the AAC descriptor offers significant reductions in training time and system resource consumption compared to high-dimensional feature sets. This makes the proposed method particularly suitable for high-throughput screening scenarios and real-time applications. As shown in Table 4, the AAC-based approach outperforms comparable methods in both predictive accuracy and efficiency, supporting its suitability for large-scale antiviral peptide screening workflows.

### 2.6. Feature Space Visualization of Physicochemical Descriptors

To further explore the separability and underlying structure of physicochemical descriptors in distinguishing NS3 protease inhibitory peptides (NS3IPs) from non-inhibitory peptides, we conducted dimensionality reduction analyses using principal component analysis (PCA), t-distributed stochastic neighbor embedding (t-SNE), and linear discriminant analysis (LDA) on the AAindex-derived feature space.

**Figure 8:**
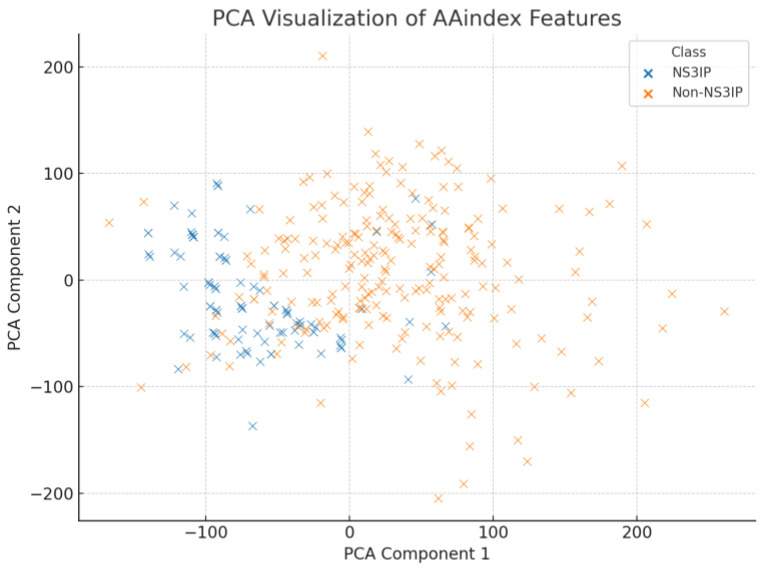
Principal component analysis (PCA) projection based on AAindex-derived physicochemical descriptors of NS3 protease inhibitory peptides (NS3IPs) and non-NS3IPs. The plot shows a partial clustering between the two classes, although some overlap remains.

**Figure 9:**
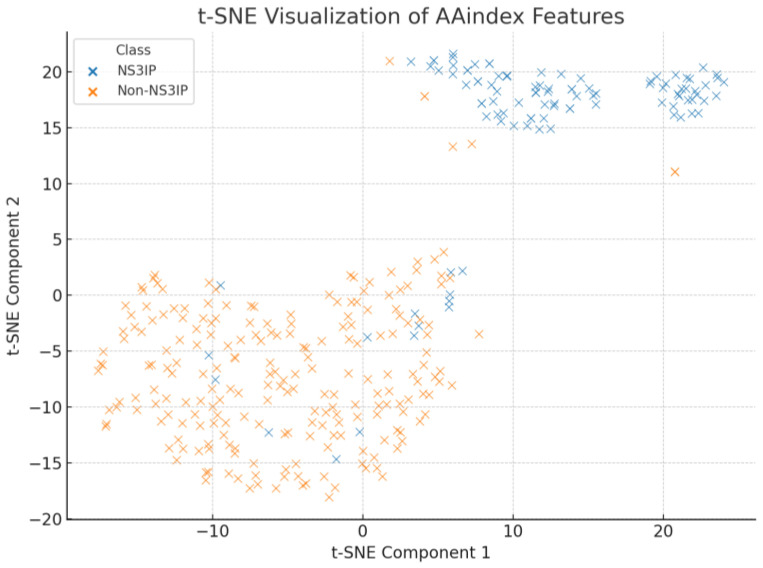
T-distributed stochastic neighbor embedding (t-SNE) projection of the AAindex-derived feature space. The plot reveals a more pronounced separation between NS3 protease inhibitory peptides (NS3IPs) and non-NS3IPs, suggesting the presence of non-linear discriminative patterns.

As shown in Figure 8, PCA revealed a moderate clustering between NS3IPs and non-NS3IPs, with some overlapping regions. This indicates that while variance exists along the principal components, a substantial portion of class information may not be captured linearly. In contrast, Figure 9 presents the t-SNE projection, which shows a more distinct separation between the two classes. This suggests that non-linear relationships among the physicochemical properties contribute significantly to their discriminative potential.

**Figure 10:**
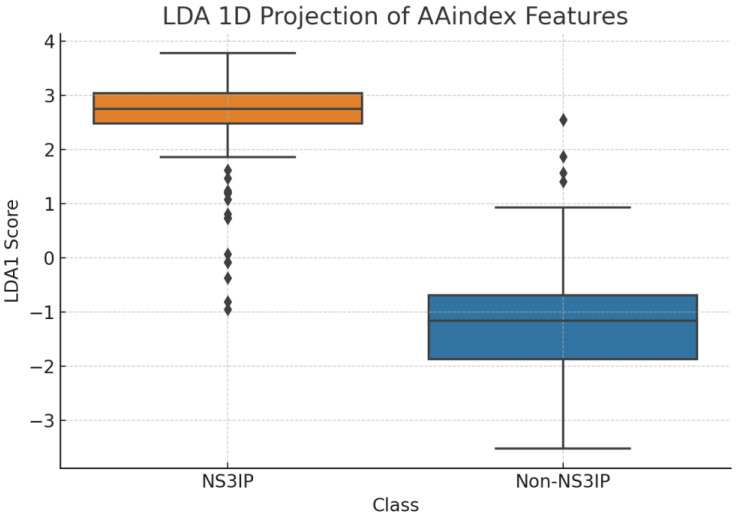
Linear discriminant analysis (LDA) projection of AAindex-derived features. The plot shows a near-linear separation between NS3 protease inhibitory peptides (NS3IPs) and non-NS3IPs, supporting the presence of class-specific physicochemical profiles relevant to NS3 protease inhibition.

Further confirmation was provided by Figure 10, where LDA yielded a near-linear separation between NS3IPs and non-inhibitory peptides. This result supports the hypothesis that AAindex-based features encode class-specific structural and chemical patterns that can be effectively leveraged by supervised learning models.

Together, these feature space visualizations complement our classification results by demonstrating that AAindex descriptors, although not selected as the final modeling feature, possess a meaningful group-level differentiation. These findings highlight the interpretive value of physicochemical profiling in understanding the molecular basis of NS3 protease inhibition.

## 3. Discussion

This study introduces a dedicated machine learning-based approach for predicting NS3 protease inhibitory peptides (NS3IPs), aiming to address the therapeutic challenges associated with drug-resistant hepatitis C virus (HCV) variants. The results demonstrate that amino acid composition (AAC), a simple yet powerful feature representation, provides a highly accurate classification performance when used in conjunction with support vector machine (SVM) and random forest (RF) models. The AAC-based models not only achieved near-perfect results during cross-validation but also maintained a 100% accuracy in independent testing, suggesting a strong generalization capability.

From a biochemical perspective, the enriched presence of hydrophobic (V, L) and positively charged residues (K, R) among NS3IPs is consistent with their potential to interact with the NS3 protease active site. These residues are commonly involved in hydrophobic and electrostatic interactions, which are essential for stable binding to enzymatic targets. The positional enrichment of lysine in the N-terminal region, as revealed by Two Sample Logo analysis, may further support the specific orientation and contact points required for effective inhibition. Similar findings have been reported in peptide-based inhibitors targeting other viral proteases, such as HIV-1 and Zika virus proteases, where conserved positively charged or hydrophobic residues mediate a strong binding affinity.

The comparative analysis of physicochemical properties also reinforces the functional distinction between NS3IPs and non-inhibitory peptides. NS3IPs demonstrated a higher net charge and lower hydrophobicity, implying a structural balance between solubility and binding affinity. These characteristics align with previous studies indicating that antiviral peptides often possess an optimized hydrophilic–hydrophobic balance for membrane interaction and target engagement.

The observed class separation in AAindex-based dimensionality reduction plots further supports the notion that inhibitory peptides exhibit conserved physicochemical patterns. These patterns, including a balanced polarity and localized charge distribution, may reflect the functional motifs necessary for protease recognition. While not directly used for model training, these descriptors contribute valuable insights into sequence–function relationships that can inform future peptide design strategies.

In terms of computational design, the observed performance of AAC—despite its low dimensionality—highlights its value as both a predictive and computationally efficient feature. While high-dimensional descriptors such as CKSAAP or DPC can offer richer representations, they increase models’ complexity and computational cost. The success of AAC in this study suggests that simpler descriptors, when properly selected and validated, may be sufficient for certain peptide classification tasks. While AAC and CKSAAP features offer a desirable efficiency and interpretability, they may not fully capture the intricate sequence–structure relationships crucial for peptide–protein interactions. To further improve the prediction accuracy and biological relevance, future work may incorporate structural modeling approaches or explore advanced deep learning techniques capable of learning complex hierarchical patterns from raw sequences.

Compared to existing antiviral peptide prediction tools such as AVPpred, AVP-IC50Pred, and Meta-IAVP, the proposed iDNS3IP platform is specifically tailored for NS3 protease inhibition. This task-specific orientation allows a more targeted feature design and model training, resulting in a higher prediction accuracy and interpretability for NS3-related antiviral discovery.

Nevertheless, certain limitations remain. The available dataset size for NS3IPs is relatively small, which may restrict the model’s exposure to broader sequence diversity and affect its generalizability. While the AAC-based models achieved a perfect performance on the independent test set, we acknowledge that the relatively small dataset size may increase the risk of overfitting. To mitigate this, 10% of the dataset was randomly partitioned at the outset and reserved as an independent test set, which was strictly excluded from model training and parameter tuning. This design ensures an objective assessment of generalization capability. Future work should therefore focus on expanding experimentally verified NS3IP datasets—through literature mining, database integration, or experimental collaboration—alongside structural modeling and deep learning–based approaches. Additionally, experimental validation—such as molecular docking simulations and in vitro assays—will be critical to substantiate the biological relevance of the predicted peptides and enhance the translational potential of the model. Furthermore, the inclusion of external validation and permutation testing will help further assess the model’s robustness.

In summary, our findings highlight the discriminative potential of simple sequence-derived features, particularly amino acid composition, in identifying NS3 protease inhibitory peptides. The observed sequence and physicochemical patterns provide useful indicators for peptide design, while the accompanying prediction tool offers a practical resource for future antiviral development targeting drug-resistant HCV strains.

## 4. Materials and Methods

### 4.1. Dataset Statistics and Data Handling

The clarity and rigor of our data-handling process are exemplified by the comprehensive distribution of NS3 protease inhibitory peptides (NS3IPs) and their non-inhibitory counterparts, as detailed in Table 1. This dataset was meticulously curated to ensure a high quality and relevance for the study, with all the included sequences being experimentally validated. Data were sourced from trusted databases, including AVPdb [32] and HIPdb [33], which provide functional annotations for inhibitory peptides, particularly those targeting the NS3 protease. These sources were selected for their specificity and reliability, making them integral to the dataset construction process. To ensure the uniqueness of sequences and eliminate redundancy, the CD-HIT tool [34] was employed to remove duplicate sequences. This step was essential for maintaining the integrity of the dataset and ensuring accurate model training. The resulting dataset comprises two classes: NS3IPs, which are peptides known to inhibit NS3 protease activity, and non-NS3IPs, which do not exhibit such activity. To facilitate an unbiased evaluation, 10% of the dataset was reserved as an independent test set, which was excluded from both the model training and parameter tuning. This segregation ensured that the final performance metrics reflect the true generalizability of the predictive model.

**Figure 11:**
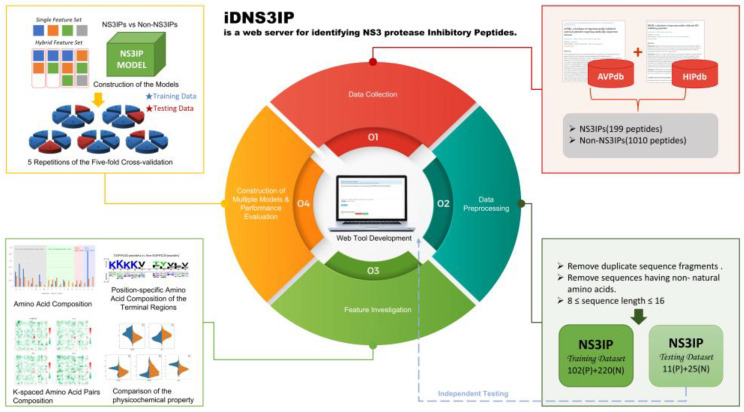
Workflow for the identification and characterization of NS3 protease inhibitory peptides (NS3IPs). The flowchart illustrates the overall process, including data collection, preprocessing, feature investigation, model construction, and performance evaluation via 5-fold cross-validation. An online prediction tool was developed for its real-time application, and independent testing was conducted to validate the model’s robustness.

The workflow of this study is illustrated in Figure 11, capturing key stages such as data collection, preprocessing, feature investigation, model construction and evaluation, independent testing, and web-based tool deployment. The data collection and preprocessing leveraged advancements in high-throughput proteomic mass spectrometry technologies, which have dramatically increased the identification of short peptides with antimicrobial and antiviral activities [35,36,37,38]. Among the numerous peptide databases available, including APD3 [39], CAMPR3 [40], DBAASP v3 [41], dbAMP [42], DRAMP 2.0 [43], and SATPdb [44], AVPdb and HIPdb were identified as particularly relevant for their detailed functional categorization of inhibitory peptides, including those targeting the NS3 protease. To ensure the data’s quality, only experimentally verified NS3IPs and non-NS3IPs were included, and redundant sequences were removed using CD-HIT. The independent test set, comprising 10% of the curated dataset, was isolated prior to model training to uphold the integrity of the evaluation process. Such rigorous data handling ensures the reliability of our model predictions and enhances their reproducibility for future research. This systematic and transparent approach to dataset construction and handling reinforces the statistical robustness of our findings. Furthermore, it underlines the practical utility of our predictive model, which has been developed to support the identification and analysis of NS3IPs as part of HCV therapeutic research.

### 4.2. Feature Investigation

To comprehensively explore sequence-derived characteristics relevant to NS3 protease inhibitory peptides (NS3IPs), we extracted multiple categories of features commonly used in the prediction of protein functions and machine learning-based modeling:

- Amino Acid Composition (AAC) [45]:

AAC represents the frequency of each of the 20 standard amino acids in a given peptide sequence. For a peptide of length L, the composition of amino acid type *i* is defined as

(1) $$f\left(i\right)=\frac{\sum x_{i}}{L}\;(1\leq i\leq 20)$$

Here, *i* represents the type of amino acid, *x_i_* denotes its frequency, and *L* is the total peptide length.

- Terminal Residue Composition (N5AAC, C5AAC) [46]:

These features represent the amino acid composition within the five residues at the N-terminus (N5AAC) and C-terminus (C5AAC) of the peptide, capturing localized sequence patterns that may influence peptide activity and binding specificity.

- Physicochemical Properties (AAindex) [31]:

A total of 544 amino acid indices were initially retrieved from the AAindex database (version 9.1). After removing indices with missing values, 531 physicochemical descriptors were retained and used to analyze potential patterns differentiating NS3IPs from non-NS3IPs.

- K-Spaced Amino Acid Pair Composition (CKSAAP) [29]:

CKSAAP estimates the frequency of amino acid pairs separated by a fixed number of residues, k. In this study, we considered four values of k: 0, 1, 2, and 3. For each peptide, the frequency of each amino acid pair (*i,j*), separated by k residues, is computed using the following formula:

(2) $$f\left(i,j\right)=\frac{\sum x_{i,j}}{L-k}\;(1\leq i,j\leq 20)$$

where $x_{i,j}$ denotes the number of occurrences of the amino acid pair (*i,j*) separated by *k* residues, and *L* is the length of the peptide sequence. These k-spaced pairwise features capture short- and medium-range residue interactions that may be critical for inhibitory activity, and they were used independently and in combination with other descriptors during models’ training and evaluation.

### 4.3. Construction of Prediction Models

To build reliable predictive models, we evaluated several machine learning algorithms, each offering unique advantages in addressing biological classification challenges:

- Support Vector Machine (SVM) [26]:

SVM, implemented with a polynomial kernel using LIBSVM, formed a cornerstone of our predictive framework. Known for its capacity to define decision boundaries in high-dimensional spaces, SVM effectively leveraged feature vectors derived from sequence-based metrics and demonstrated a strong performance in peptide classification tasks.

- Random Forest (RF) [27]:

As an ensemble learning method, RF constructs multiple decision trees during training and aggregates their predictions to determine the final output. Its robustness against overfitting, ability to handle missing data, and provision of feature importance rankings made RF a valuable tool in our study.

- K-Nearest Neighbors (KNN) [47]:

KNN classifies data points based on their proximity to labeled neighbors. Its non-parametric nature and adaptability to diverse data distributions rendered it a versatile option for both classification and regression problems.

- Decision Tree (DT) [48]:

DTs organize data into a hierarchical tree structure, recursively partitioning the dataset into subsets with specific outcomes. While highly interpretable, DTs can be prone to overfitting without appropriate regularization or pruning strategies.

- AdaBoost [49]:

This boosting algorithm iteratively improves models’ performance by focusing on previously misclassified samples. AdaBoost’s adaptability and ensemble approach make it effective for enhancing classification robustness.

After rigorous evaluation, SVM and RF emerged as the most effective algorithms, achieving superior accuracy and reliability in predicting NS3IPs. These models were subsequently selected for further evaluation and deployment.

### 4.4. Performance Evaluation

The predictive performance of the NS3IP models was assessed using five repetitions of five-fold cross-validation on the training dataset. The models were implemented using the LIBSVM framework for support vector machines (SVMs) and evaluated with multiple classification metrics. These included true positives (TPs), false negatives (FNs), true negatives (TNs), and false positives (FPs). The performance metrics are defined as follows:

- *Sensitivity* (*Sn*): Measures the proportion of NS3IPs correctly identified.

(3) $$Sensitivity\;(Sn)=\frac{TP}{TP+FN}$$

- *Specificity* (*Sp*): Measures the proportion of non-NS3IPs correctly identified.

(4) $$Specificity\;(Sp)=\frac{TN}{TN+FP}$$

- *Accuracy* (*Acc*): Measures the overall proportion of correctly classified peptides.

(5) $$Accuracy\;(Acc)=\frac{TP+TN}{TP+FP+TN+FN}$$

- *Balanced Accuracy* (*B.ACC*): Provides an average of sensitivity and specificity, which is particularly important when handling imbalanced datasets.

(6) $$Balanced\;Accuracy\;\left(B.ACC\right)=\frac{Sensitivity+Specificity}{2}$$

- *Matthews Correlation Coefficient* (*MCC*): A comprehensive metric accounting for all four confusion matrix categories, particularly effective for imbalanced datasets.

(7) $$\begin{array}{l} Matthews\;Correlation\;Coefficient\;(MCC)\\ \;\;\;\;\;\;\;\;\;\;\;\;\;\;\;\;\;\;\;\;\;\;\;\;\;=\frac{\left(TP\times TN\right)-\left(FP\times FN\right)}{\sqrt{\left(TP+FP\right)\left(TP+FN\right)\left(TN+FP\right)\left(TN+FN\right)}} \end{array}$$

The use of balanced accuracy plays a key role in mitigating bias due to class imbalance, where NS3IPs may constitute a minority compared to non-inhibitory peptides [50]. Unlike traditional accuracy, B.ACC integrates both sensitivity and specificity, offering a more equitable performance assessment across classes.

In addition, the Matthews correlation coefficient (MCC) serves as a robust single-value measure that reflects both a positive and negative classification performance, enhancing the reliability of the model for real-world applications in peptide prediction.

## 5. Conclusions

This study introduces a robust and efficient framework for predicting NS3 protease inhibitory peptides (NS3IPs), representing the first computational model specifically tailored for this critical therapeutic target in hepatitis C virus (HCV) therapy. Leveraging amino acid composition (AAC) as the primary feature set and employing support vector machine (SVM) and random forest (RF) classifiers, our models achieved an outstanding predictive performance, including perfect scores across independent testing metrics such as sensitivity, specificity, and accuracy. The simplicity and computational efficiency of the 20-dimensional AAC feature set make it particularly well suited for large-scale and real-time peptide screening, addressing a pressing need in resource-limited settings. To translate computational insights into practical applications, we developed iDNS3IP, an online prediction tool that integrates both SVM and RF classifiers. This user-friendly platform enables researchers to make real-time predictions of NS3IP inhibitory potential, offering flexibility and accessibility through an intuitive interface. By combining a high prediction accuracy with ease of use, iDNS3IP positions itself as a valuable resource for accelerating the discovery of peptide inhibitors and advancing the development of antiviral drugs. The novelty of this work lies not only in its focus on NS3 protease, a pivotal enzyme in the HCV life cycle, but also in demonstrating how computational approaches can address the limitations of traditional drug development. Comparative analyses highlight the strengths of our AAC-based models, which effectively balance predictive accuracy and computational cost, providing a practical and scalable solution for the identification of peptide inhibitors. These contributions establish a foundation for expanding computational antiviral research beyond HCV to other viral protease targets. Looking ahead, this study opens avenues for further innovation. Integrating additional sequence-derived features, such as K-spaced amino acid pairs (CKSAAP) or advanced physicochemical properties, could enhance the model’s performance. Moreover, deep learning techniques hold promise for uncovering complex sequence patterns and improving the predictive accuracy. Crucially, experimental validation of the predicted NS3IPs will be essential to confirm their inhibitory potential and facilitate their development into therapeutic agents. By addressing key challenges in HCV drug resistance and extending its framework to other viral targets, this study demonstrates the transformative potential of specialized computational models. It sets the stage for future innovations in the discovery of antiviral peptides, contributing significantly to the development of next-generation therapies.

## Figures and Tables

**Table 1:** Dataset composition and sample counts for training and independent testing.

Dataset	Number of NS3IPs	Number of Non-NS3IPs
Raw dataset	199	1010
Training dataset	102	220
Independent test set	11	25

**Table 2:** Performance of NS3IP prediction models trained on individual feature sets, evaluated using five repetitions of 5-fold cross-validation.

Classifier	Feature	Sensitivity (%)	Specificity (%)	Accuracy (%)	B.ACC (%)	MCC
SVM	AAC	95.10 ± 0.0098	99.60 ± 0.0014	98.85 ± 0.0012	97.35 ± 0.0044	0.96 ± 0.0043
	N5AAC	92.94 ± 0.0107	96.82 ± 0.0091	95.59 ± 0.0056	94.88 ± 0.0052	0.90 ± 0.0124
	C5AAC	91.18 ± 0.0120	98.64 ± 0.0085	96.27 ± 0.0054	94.91 ± 0.0056	0.91 ± 0.0127
	DPC	96.86 ± 0.0107	99.55 ± 0.0000	98.70 ± 0.0034	98.20 ± 0.0054	0.97 ± 0.0079
	C1SAAP	95.49 ± 0.0088	99.64 ± 0.0020	98.32 ± 0.0035	97.56 ± 0.0049	0.96 ± 0.0082
	C2SAAP	95.88 ± 0.0082	99.91 ± 0.0020	98.63 ± 0.0035	97.90 ± 0.0047	0.97 ± 0.0082
	C3SAAP	94.31 ± 0.0082	99.73 ± 0.0041	98.01 ± 0.0028	97.02 ± 0.0036	0.95 ± 0.0065
	AAindex	94.51 ± 0.0203	97.45 ± 0.0025	96.52 ± 0.0067	95.98 ± 0.0103	0.92 ± 0.0159
RF	AAC	93.92 ± 0.0128	99.64 ± 0.0050	97.83 ± 0.0066	96.78 ± 0.0081	0.95 ± 0.0154
	N5AAC	91.96 ± 0.0082	97.82 ± 0.0075	95.96 ± 0.0066	94.89 ± 0.0066	0.91 ± 0.0152
	C5AAC	93.73 ± 0.0246	97.00 ± 0.0089	95.96 ± 0.0101	95.36 ± 0.0132	0.91 ± 0.0234
	DPC	94.12 ± 0.0139	99.36 ± 0.0041	97.70 ± 0.0042	96.74 ± 0.0064	0.95 ± 0.0096
	C1SAAP	92.55 ± 0.0149	99.36 ± 0.0025	97.20 ± 0.0058	95.96 ± 0.0082	0.94 ± 0.0135
	C2SAAP	91.57 ± 0.0112	99.36 ± 0.0025	96.89 ± 0.0044	95.47 ± 0.0061	0.93 ± 0.0102
	C3SAAP	91.37 ± 0.0082	99.45 ± 0.0020	96.89 ± 0.0038	95.41 ± 0.0050	0.93 ± 0.0089
	AAindex	86.27 ± 0.0069	98.36 ± 0.0061	94.53 ± 0.0052	92.32 ± 0.0052	0.87 ± 0.0125

**Table 3:** Performance of NS3IP prediction models trained on individualhybrid feature sets, evaluated using five repetitions of 5-fold cross-validation.

Classifier	Feature	Sensitivity (%)	Specificity (%)	Accuracy (%)	B.ACC (%)	MCC
SVM	AAC + DPC	97.45 ± 0.0088	99.55 ± 0.0000	98.88 ± 0.0028	98.50 ± 0.0044	0.97 ± 0.0064
	N5AAC + C5AAC	92.75 ± 0.0054	98.73 ± 0.0038	96.83 ± 0.0014	95.74 ± 0.0014	0.93 ± 0.0033
	AAC + DPC + N5AAC + C5AAC	94.71 ± 0.0112	99.64 ± 0.0020	98.07 ± 0.0026	97.17 ± 0.0048	0.96 ± 0.0059
	AAC + DPC+ CKSAAP	97.25 ± 0.0082	99.36 ± 0.0025	98.70 ± 0.0034	98.31 ± 0.0045	0.97 ± 0.0079
RF	AAC + DPC	92.16 ± 0.0155	99.73 ± 0.0025	97.33 ± 0.0052	95.94 ± 0.0078	0.94 ± 0.0120
	N5AAC + C5AAC	95.10 ± 0.0120	98.91 ± 0.0025	97.70 ± 0.0035	97.00 ± 0.0057	0.95 ± 0.0082
	AAC + DPC + N5AAC + C5AAC	93.14 ± 0.0069	99.55 ± 0.0000	97.52 ± 0.0022	96.34 ± 0.0035	0.94 ± 0.0051
	AAC + DPC+ CKSAAP	93.14 ± 0.0098	99.73 ± 0.0025	97.64 ± 0.0035	96.43 ± 0.0051	0.95 ± 0.0082

**Table 4:** Comparison of independent test set performance between our proposed method and existing prediction tools.

Method	Sensitivity (%)	Specificity (%)	Accuracy (%)	B.ACC (%)	MCC
iDNS3IP (SVM)	100	100	100	100	1
iDNS3IP (RF)	100	100	100	100	1
AVP-IC50Pred	100	0	30.56	50	0

## Data Availability

The dataset supporting this study is openly available at http://mer.hc.mmh.org.tw/iDNS3IP/dataset.php.

## Supplementary Materials

The following supporting information can be downloaded at: https://www.mdpi.com/article/10.3390/ijms26115356/s1.

## Abbreviations

The following abbreviations are used in this manuscript:

AAC	Amino Acid Composition
AAindex	Physicochemical Properties (Amino Acid Index)
AUC	Area Under the Curve
B.ACC	Balanced Accuracy
CKSAAP	Composition of K-Spaced Amino Acid Pairs
C5AAC	C-Terminal Five-Residue Amino Acid Composition
DAAs	Direct-Acting Antivirals
DPC	Dipeptide Composition
DT	Decision Tree
HCV	Hepatitis C Virus
KNN	K-Nearest Neighbors
LDA	Linear Discriminant Analysis
MCC	Matthews Correlation Coefficient
NS3IPs	NS3 Protease Inhibitory Peptides
N5AAC	N-terminal Five-Residue Amino Acid Composition
PCA	Principal Component Analysis
RF	Random Forest
ROC	Receiver Operating Characteristic
SVM	Support Vector Machine
t-SNE	T-Distributed Stochastic Neighbor Embedding
