# Supplementary material for: iDNS3IP: Identification and Characterization of HCV NS3 Protease Inhibitory Peptides
Source: Int J Mol Sci. 2025 Jun 3;26(11):5356. doi: 10.3390/ijms26115356 (PMC12154261; doi:10.3390/ijms26115356)
Supplement: Supplementary file 1 [file ijms-26-05356-s001.zip › Supplementary Data/Supplementary File Legends.pdf]

Supplementary File Legends

Table S1. The results of five repetitions of 5-fold cross-validation of the NS3IP prediction models trained with individual feature sets using various machine learning methods. Metrics include sensitivity, specificity, accuracy, balanced accuracy (B.Accuracy), and Matthews correlation coefficient (MCC).

Table S2. The results of five repetitions of 5-fold cross-validation of the NS3IP prediction models trained with hybrid feature sets using various machine learning methods. Metrics include sensitivity, specificity, accuracy, balanced accuracy (B.Accuracy), and Matthews correlation coefficient (MCC).
