# Supplementary material for: iDNS3IP: Identification and Characterization of HCV NS3 Protease Inhibitory Peptides
Source: Int J Mol Sci. 2025 Jun 3;26(11):5356. doi: 10.3390/ijms26115356 (PMC12154261; doi:10.3390/ijms26115356)
Supplement: Supplementary file 1 [file ijms-26-05356-s001.zip › Supplementary Data/Supplementary_Table_S1.pdf]

Table S1. The results of five repetitions of 5-fold cross-validation of the NS3IP prediction models trained with individual feature sets using various machine learning methods. Metrics include sensitivity, specificity, accuracy, balanced accuracy (B.Accuracy), and Matthews correlation coefficient (MCC).

Classifier	Feature	Sensitivity (%)	Specificity (%)	Accuracy (%)	B.Accuracy (%)	MCC
SVM	AAC	95.10±0.0098	99.60±0.0014	98.85±0.0012	97.35±0.0044	0.96± 0.0043
	N5AAC	92.94±0.0107	96.82±0.0091	95.59±0.0056	94.88±0.0052	0.90± 0.0124
	C5AAC	91.18±0.0120	98.64±0.0085	96.27±0.0054	94.91±0.0056	0.91± 0.0127
	DPC	96.86±0.0107	99.55±0.0000	98.70±0.0034	98.20±0.0054	0.97± 0.0079
	C1SAAP	95.49±0.0088	99.64±0.0020	98.32±0.0035	97.56±0.0049	0.96± 0.0082
	C2SAAP	95.88±0.0082	99.91±0.0020	98.63±0.0035	97.90±0.0047	0.97± 0.0082
	C3SAAP	94.31±0.0082	99.73±0.0041	98.01±0.0028	97.02±0.0036	0.95± 0.0065
	AAindex	94.51±0.0203	97.45±0.0025	96.52±0.0067	95.98±0.0103	0.92± 0.0159
RF	AAC	93.92±0.0128	99.64±0.0050	97.83±0.0066	96.78±0.0081	0.95± 0.0154
	N5AAC	91.96±0.0082	97.82±0.0075	95.96±0.0066	94.89±0.0066	0.91± 0.0152
	C5AAC	93.73±0.0246	97.00±0.0089	95.96±0.0101	95.36±0.0132	0.91± 0.0234

	DPC	94.12±0.0139	99.36±0.0041	97.70±0.0042	96.74±0.0064	0.95±0.0096
	C1SAAP	92.55±0.0149	99.36±0.0025	97.20±0.0058	95.96±0.0082	0.94±0.0135
	C2SAAP	91.57±0.0112	99.36±0.0025	96.89±0.0044	95.47±0.0061	0.93±0.0102
	C3SAAP	91.37±0.0082	99.45±0.0020	96.89±0.0038	95.41±0.0050	0.93±0.0089
	AAindex	86.27±0.0069	98.36±0.0061	94.53±0.0052	92.32±0.0052	0.87±0.0125
KNN	AAC	89.22±0.0000	95.09±0.0020	93.23±0.0014	92.15±0.0010	0.84±0.0030
	N5AAC	83.33±0.0120	91.64±0.0061	89.01±0.0035	87.48±0.0048	0.75±0.0082
	C5AAC	86.67±0.0112	91.27±0.0038	89.81±0.0046	88.97±0.0061	0.77±0.0109
	DPC	100.00±0.0000	09.82±0.0178	38.39±0.0121	54.91±0.0089	0.18±0.0173
	C1SAAP	100.00±0.0000	04.18±0.0099	34.53±0.0067	52.09±0.0049	0.12±0.0143
	C2SAAP	99.22±0.0082	32.27±0.0216	53.48±0.0154	65.74±0.0122	0.35±0.0206
	C3SAAP	100.00±0.0000	20.82±0.0180	45.90±0.0123	60.41±0.0090	0.28±0.0139

	AAindex	87.06±0.0128	96.18±0.0025	93.29±0.0047	91.62±0.0068	0.84±<br>0.0112
DT	AAC	91.76±0.0256	97.64±0.0177	95.78±0.0127	94.70±0.0136	0.90±<br>0.0287
	N5AAC	87.84±0.0226	95.36±0.0075	92.98±0.0047	91.60±0.0088	0.84±<br>0.0116
	C5AAC	89.80±0.0236	94.45±0.0059	92.98±0.0081	92.13±0.0118	0.84±<br>0.0194
	DPC	93.53±0.0149	98.73±0.0020	97.08±0.0047	96.13±0.0074	0.93±<br>0.0110
	C1SAAP	87.84±0.0226	97.64±0.0075	94.53±0.0042	92.74±0.0085	0.87±<br>0.0097
	C2SAAP	89.61±0.0088	96.73±0.0104	94.47±0.0071	93.17±0.0061	0.87±<br>0.0162
	C3SAAP	88.82±0.0054	97.55±0.0076	94.78±0.0040	93.18±0.0025	0.88±<br>0.0095
	AAindex	87.25±0.0219	92.18±0.0109	90.62±0.0127	89.72±0.0147	0.79±<br>0.0290
AdaBoost	AAC	91.57±0.0132	97.27±0.0056	95.47±0.0061	94.42±0.0075	0.89±<br>0.0142
	N5AAC	86.67±0.0149	97.36±0.0087	93.98±0.0078	92.02±0.0088	0.86±<br>0.0184
	C5AAC	80.39±0.0139	95.09±0.0075	90.43±0.0067	87.74±0.0079	0.78±<br>0.0159

DPC	86.67±0.0191	99.55±0.0000	95.47±0.0061	93.11±0.0096	0.90± 0.0138
C1SAAP	82.35±0.0139	96.64±0.0041	92.11±0.0035	89.49±0.0060	0.82± 0.0084
C2SAAP	84.90±0.0164	95.18±0.0025	91.93±0.0062	90.04±0.0089	0.81± 0.0149
C3SAAP	73.33±0.0234	99.36±0.0025	91.12±0.0061	86.35±0.0107	0.80± 0.0133
AAindex	89.61±0.0256	94.91±0.0189	93.23±0.0080	92.26±0.0077	0.84± 0.0173
