# Supplementary material for: iDNS3IP: Identification and Characterization of HCV NS3 Protease Inhibitory Peptides
Source: Int J Mol Sci. 2025 Jun 3;26(11):5356. doi: 10.3390/ijms26115356 (PMC12154261; doi:10.3390/ijms26115356)
Supplement: Supplementary file 1 [file ijms-26-05356-s001.zip › Supplementary Data/Supplementary_Table_S2.pdf]

Table S2. The results of five repetitions of 5-fold cross-validation of the NS3IP prediction models trained with hybrid feature sets using various machine learning methods. Metrics include sensitivity, specificity, accuracy, balanced accuracy (B.Accuracy), and Matthews correlation coefficient (MCC).

Classifier	Feature	Sensitivity (%)	Specificity (%)	Accuracy (%)	B.Accuracy (%)	MCC
SVM	AAC+DPC	97.45±0.0088	99.55±0.0000	98.88±0.0028	98.50±0.0044	0.97±0.0064
	N5AAC+C5AAC	92.75±0.0054	98.73±0.0038	96.83±0.0014	95.74±0.0014	0.93±0.0033
	AAC+DPC+ N5AAC+C5AAC	94.71±0.0112	99.64±0.0020	98.07±0.0026	97.17±0.0048	0.96±0.0059
	AAC+DPC+ CKSAAP	97.25±0.0082	99.36±0.0025	98.70±0.0034	98.31±0.0045	0.97±0.0079
RF	AAC+DPC	92.16±0.0155	99.73±0.0025	97.33±0.0052	95.94±0.0078	0.94±0.0120
	N5AAC+C5AAC	95.10±0.0120	98.91±0.0025	97.70±0.0035	97.00±0.0057	0.95±0.0082
	AAC+DPC+ N5AAC+C5AAC	93.14±0.0069	99.55±0.0000	97.52±0.0022	96.34±0.0035	0.94±0.0051
	AAC+DPC+ CKSAAP	93.14±0.0098	99.73±0.0025	97.64±0.0035	96.43±0.0051	0.95±0.0082
KNN	AAC+DPC	99.80±0.0044	30.36±0.0237	52.36±0.0155	65.08±0.0108	0.35±0.0148
	N5AAC+C5AAC	89.02±0.0082	94.27±0.0025	92.61±0.0040	91.65±0.0051	0.83±0.0095
	AAC+DPC+ N5AAC+C5AAC	99.41±0.0054	40.73±0.0114	59.32±0.0093	70.07±0.0082	0.42±0.0141
	AAC+DPC+ CKSAAP	100.00±0.0000	07.73±0.0161	36.96±0.0110	53.86±0.0080	0.16±0.0177
DT	AAC+DPC	93.53±0.0132	98.00±0.0052	96.58±0.0058	95.76±0.0074	0.92±0.0135
	N5AAC+C5AAC	90.59±0.0236	96.64±0.0041	94.72±0.0076	93.61±0.0117	0.88±0.0182
	AAC+DPC+ N5AAC+C5AAC	93.92±0.0128	98.09±0.0020	96.77±0.0047	96.01±0.0068	0.93±0.0110
	AAC+DPC+ CKSAAP	92.35±0.0145	97.91±0.0041	96.15±0.0028	95.13±0.0057	0.91±0.0066
AdaBoost	AAC+DPC	91.96±0.0213	98.55±0.0059	96.46±0.0056	95.25±0.0093	0.92±0.0132
	N5AAC+C5AAC	88.43±0.0244	98.09±0.0081	95.03±0.0049	93.26±0.0094	0.88±0.0116
	AAC+DPC+ N5AAC+C5AAC	92.75±0.0054	98.64±0.0056	96.77±0.0035	95.69±0.0031	0.93±0.0082
	AAC+DPC+ CKSAAP	92.55±0.0132	98.55±0.0075	96.65±0.0077	95.55±0.0087	0.92±0.0180
